# A retrospective cohort study to predict severe dengue in Honduran patients

**DOI:** 10.1186/s12879-017-2800-3

**Published:** 2017-10-11

**Authors:** Eduardo Fernández, Marek Smieja, Stephen D. Walter, Mark Loeb

**Affiliations:** 1 Department of Clinical Epidemiology and Biostatistics, McMaster University, Hamilton, ON Canada; 2 Department of Pathology and Molecular Medicine, Institute for Infectious Disease Research McMaster University, Hamilton, ON Canada

**Keywords:** Severe dengue, Predictors, Models

## Abstract

**Background:**

An important challenge in the identification of dengue is how to predict which patients will go on to experience severe illness, which is typically characterized by fever, thrombocytopenia, haemorrhagic manifestations, and plasma leakage. Accurate prediction could result in the appropriate hospital triage of high risk patients. The objective of this study was to identify clinical factors observed within the first 24 h of hospital admission that could predict subsequent severe dengue.

**Methods:**

We conducted a retrospective cohort study of 320 patients with febrile illness who had confirmation of dengue within one week of admission, using data from the 2009–2010 Honduras Epidemiological Survey for Dengue. The outcome measure was plasma leakage defined using hemoconcentration ≥15% as determined by serial hematocrit testing. We conducted univariable analysis and multivariable logistic regression analysis to construct a predictive model for severe dengue.

**Results:**

Thirty-four (10.6%) of patients in the 320 patient cohort had hemoconcentration ≥15%. In the final multivariable logistic regression model the presence of ascites, OR 7.29, 95% CI 1.85 to 28.7, and a platelet count <50,000 platelets/mm^3^ at admission, OR 3.02, 95% CI 1.42 to 6.42, were significantly associated with plasma leakage, while the presence of petechiae, OR 0.24 95% CI 0.080 to 0.73, and headache, OR 0.38, 95% CI 0.15 to 0.95, were negatively associated with leakage. Using an estimated probability of 7% as a threshold for a person being considered a severe case correctly predicted 26 of the 34 severe cases (sensitivity 76.4%) and 201 of the 286 non-severe cases (specificity of 70.3%) for a percentage correctly classified of 70.9%.

**Conclusion:**

We identified signs and symptoms that can correctly identify a majority of patients who eventually develop severe dengue in Honduras. It will be important to further refine our models and validate them in other populations.

## Background

Dengue fever is a mosquito-borne disease, with a high incidence in tropical and subtropical regions; most cases are reported in South East Asia, the Americas Region and the Pacific Basin [1]. Treatment for the disease is still in development and one vaccine is currently being marketed in affected countries with several other candidates being tested”.

Identifying cases of dengue that are likely to eventually develop severe dengue is an important challenge in endemic regions. Previous studies have had varied success in identifying patients that go on to develop severe illness [2–12]. Limitations of these studies include lack of validation [7], single site study [4, 8–10], lack of adjustment for confounding variables [4], restricted to children [8, 11], and selection bias [12]. Although a diagnosis of dengue can be confirmed within days of clinical presentation, identifying those patients who will go on to develop severe dengue could serve as an important form of triage for hospitals. That is, patients at low risk would not require the intensive follow up of high risk patients and this could help reduce the burden on the healthcare system. Conversely, high risk patients would be monitored more closely and given early supportive care as they develop evidence of severe disease. Symptoms and signs are typically recorded for patients with suspected dengue and widely available testing, such as blood platelets and hematocrit, is usually done. Thus, a combination of early symptoms and laboratory tests could help quantify the likelihood of severe dengue disease.

Using data from Honduras, the objective of this study was to identify clinical and laboratory factors found at admission that predict severe dengue, previously known as dengue hemorrhagic fever (DHF) and dengue shock syndrome (DSS), in patients that subsequently had a confirmed diagnosis of dengue.

## Methods

### Study design

We conducted a retrospective cohort study using clinical and laboratory data from Honduras Ministry of Health to predict severe dengue. Data on signs and symptoms of patients who present with dengue is routinely collected in Honduras and submitted to the Honduran National Classification Committee for classification of cases.

### Setting and participants

We first used the Ministry of Health data to identify patients with confirmed dengue. Data was collected between 2009 and 2010 from patients who presented with fever and ≥2 symptoms to one of 5 hospitals and outpatient clinics located in Tegucigalpa and San Pedro Sula (the two largest cities in Honduras) and were confirmed to have dengue by serology or viral isolation. If a patient presented within 5 days of the first symptom onset, samples were processed for viral culture following guidelines from the U.S Centers for Disease Control and Prevention (CDC) [13]. If the blood sample was taken on the sixth or later day of onset of symptoms, a test for antibody detection was done using IgM antibody-capture enzyme-linked immunosorbent (IgM Mac ELISA).

### Data sources

Serology and viral isolation were performed at the Honduras National Laboratory of Virology according to CDC guidelines and standardized techniques. General practitioners, nurses, and microbiologists completed a data collection form which included epidemiological and clinical information. Symptoms and signs were collected at first presentation while hematology and biochemical tests recorded were those at presentation and those repeated approximately one week later. Fever was defined as a temperature of >37.5 degrees Celsius. Both objective measurements of fever and self-report of fever were included.

### Study size

This cohort consists of confirmed dengue patients who were evaluated for severe dengue in tertiary care hospitals in Tegucigalpa and San Pedro Sula. Of the 320 cases, 315 (98%) were diagnosed with dengue by IgM and five (2%) were diagnosed by viral isolation. The patients in this dataset had at least two consecutive readings of hematocrit and platelets in the first 24 h after their admission to the hospital.

### Predictor variables

We considered a number of predictor variables for this analysis including 1) demographics, such as age (considered as a categorical variable where children were defined ≤12 years) and sex, 2) symptoms reported within the first 24 h of admission, including fever, headache, retroocular pain, arthralgia, myalgia, rash, anorexia and vomiting, 3) bleeding manifestations, including petechiae, ecchymosis, hematemesis, melena, positive tourniquet, epistaxis, gingival bleeding, tourniquet test, hematuria, metrorrhagia, 4) symptoms and signs of capillary leakage, including abdominal pain, cold extremities, sweating, pale skin, pericardial and pleural effusions, ascites, as well as reduction of medial arterial pressure (based on blood pressure recorded within the first 24 h of admission).

Laboratory data on leukocyte count, platelet count, hemoglobin, and the hematocrit, collected upon admission and 24 h later, were also used as predictor variables. Thrombocytopenia was defined as a platelet count of 100,000 platelets/mm^3^ or less [13]. We considered platelets as a binary variable using a cut-off value of 50,000 platelets/mm^3^ at 24 h and a cut-off of 100,000 platelets/mm^3^ at 48 h.

### Outcome

The outcome or dependent variable was plasma leakage. Although death is the most serious outcome for severe dengue, it is uncommon in the Americas [13, 14]. Plasma leakage is a central event in the pathogenesis of severe dengue, leading to hypovolemia, hypotension, edema, shock, and potentially death [1, 13].

Key elements in the classification of severe dengue include the presence of plasma leakage manifestations and hemorrhagic symptoms. In our study, plasma leakage was measured by hemoconcentration using two consecutive readings of hematocrit, the first hematocrit taken at admission and the second 24 h later. Hemoconcentration gradually increases over the course of severe dengue. Although a threshold of 20% above baseline is frequently used, we selected a threshold of 15% to increase the sensitivity of the analysis [7, 14]. Previous studies in Colombia and Puerto Rico have considered thresholds as low as 10% of hemoconcentration, having found that sensitivity is increased while specificity is not significantly reduced [15, 16]. We reasoned that such an intermediate value would maximize the number of cases identified while maintaining specificity.

### Data analysis

We conducted a univariate analysis assessing the relationship between the predictor variables and plasma leakage as defined by a 15% increase in hematocrit, an indicator of severe dengue. Variables with a *p* value <0.2 were considered for multivariable analysis using logistic regression built using forward step-wise selection [17]. Variables were kept in the final model if there were statistically significant (*p* < 0.05) and if no collinearity was observed among them. We assessed collinearity using the tolerance statistic (values >0.2 were acceptable) and the variance inflation factor (VIF), which was acceptable if values were <5. Goodness of fit was determined using Hosmer-Lemeshow statistics (*p* > 0.05 for the model). The final model was validated internally using the bootstrap technique (by sampling with replacement using 320 individuals and sampling 1000 times**)** [18].

Using the equation of the final logistic regression model, the frequency distribution of predicted probabilities of severer dengue was assessed in order to create a threshold defining the severe case and non-severe case assignments. We selected a threshold for the probability of being a case so that both sensitivity and specificity were maximized. We created an ROC graph to verify the sensitivity and specificity for the selected threshold.

Additionally, as a sensitivity analysis, we created a dichotomous variable for the type of health care facility where patients had presented to either the social security system (accessible only to the insured employed population) or at public health clinics (open to the general population). This was done to account for potential centre effect, that is, differences between the two patient populations.

The analysis was conducted using SPSS version 18.

## Results

### Participants and main results

Of the 320 patients in the cohort, 139 (43.4%) were female and the mean age was 22.4 years. Two hundred and forty-four (49.2%) were hospitalized six or more days after the onset of the first symptom. Thirty-four (10.6%) met our definition of plasma leakage, based on a hemoconcentration of ≥15%. In univariable analysis, cases were less likely to have petechiae (odds ratio [OR] 0.27 (0.09 to 0.78)) but were more likely to have ascites (OR 5.31, 95% CI 1.47 to 19.2) and platelets ≤50,000 mm^3^, OR 2.95 (95% CI 1.38 to 6.74) (Table 1).

**Table 1:** Results of the univariate and multivariable (adjusted) analysis of predictors for severe dengue in a cohort of 340 patients presenting to hospitals in Honduras

	Cases *N* = 34	Non-cases *N* = 286	Odds ratio (95% CI)	Adjusted Odds ratio (95% CI)
Ascites	4 (11.76%)	7 (2.44%)	5.31 (1.47 to 19.2)	7.29 (1.85 to 28.7)
Headache	26 (76.47%)	252 (88.11%)	0.44 (0.18 to 1.05)	0.38 (0.15 to 0.95)
Skin rash	9 (26.47%)	113 (39.51%)	0.55 (0.25 to 1.22)	
Occular pain	23 (67.64%)	221 (77.27%)	0.62 (0.28 to 1.32)	
Petechiae	4 (11.76%)	95 (33.21%)	0.27 (0.09 to 0.78)	0.24 (0.08 to 0.73)
Ecchymosis	0(1.47%)	35 (12.4%)	0.10(0.006 to 1.76)	
Sex (male)	21 (61.76%)	156 (54.54%)	1.34 (0.65 to 2.80)	
Age (≥12 years)	20 (58.82%)	173 (60.49%)	0.93 (0.45 to 1.96)	
Symptom onset to admission (≤ 3 days)	5 (14.709%)	64 (22.38%)	0.60 (0.20 to 1.53)	
Platelets at admission (≤50,000)	24 (70.58%)	128 (44.76%)	2.95 (1.38 to 6.74)	2.9 (1.42 to 6.42)
Platelets at 48 h (≤100,000)	1 (2.94%)	31 (10.84%)	0.25 (0.01 to 1.39)	
Fever	34 (100%)	282 (98.6%)	0.99(0.97 to 1.00)	
Arthralgia	26 (76.5%)	220 (77.0%)	0.97 (0.42 to 2.25)	
Myalgia	29 (85.3%)	251(87.8%)	0.81 (0.30 to 2.22)	
Anorexia	27 (79.4%)	233 (81.4%)	0.88 (0.36 to 2.12)	
Vomiting	15 (44.1%)	98 (34.3%)	1.50 (0.74 to 3.11)	
Hematemesis	1 (2.9%)	20(7.0%)	0.40 (0.05 to 3.10)	
Melena	2 (5.9%)	7 (2.4%)	2.50 (0.50 to 12.50)	
Tourniquet test	3 (8.8%)	15 (5.2%)	1.75 (0.48 to 6.38)	
Epistaxis	7 (20.6%)	43 (15.0%)	1.46(0.60 to 3.58)	
Gingival bleeding	3 (8.8%)	16 (5.6%)	1.63 (0.45 to 5.90)	
Hematuria	1 (2.9%)	9 (3.2%)	0.93 (0.11 to 7.60)	
Methrorragia	1 (2.9%)	8(2.8%)	1.05 (0.13 to 8.69)	
Abdominal Pain	16 (47.0%)	128 (4.5%)	1.10 (0.54 to 2.29)	
Cold extremity	10 (29.4%)	97 (33.9%)	0.81 (0.37 to 1.77)	
Sweating	14 (41.2%)	137 (47.9%)	0.76 (0.37 to 1.60)	
Pallor (skin)	15 (44.1%)	118 (41.3%)	1.12 (0.55 to 2.30)	
Pericardium effusion	0 (0%)	4 (1.4%)	0.98 (0.97 to 1.00)	
Pleural effusion	2 (5.9%)	8 (2.8%)	2.20 (0.44 to 10.70)	

The following variables were considered for multivariable analysis: ascites, petechiae, ecchymosis, headache, skin rash, retro-ocular pain, platelet count on admission. As specified a priori, age and sex were included in the model. The final model included the variables headache OR 0.38, 95% CI 0.15 to 0.95, petechiae OR 0.24 95% CI 0.08 to 0.73, ascites OR 7.29, 95% CI 1.85 to 28.7, and platelets <50,000 platelets/mm^3,^ OR 3.02, 95% CI 1.42 to 6.42 at baseline (Table 2).

**Table 2:** Results of the bootstrap for the final model^a^

Predictor	B^b^	Bias^c^	SE	P	B 95% CI
Ascites	1.99	−.182	−2.49	.001	0.37 to 3.45
Petechiae	−1.42	−.419	2.60	.006	3.22 to 0.57
Platelets at admission	1.09	.007	0.43	.008	0.27 to 1.98
Headache	0.98	0.04	0.55	.041	−1.95 to 0.26

^a^Data on 320 participants; 1000 replications

^b^B refers to average of the bootstrap estimates

^c^The bias estimates were compared to the standard error of values of the predictors of the subsamples

For goodness of fit, the Hosmer-Lemeshow test showed that the observed event frequencies matched the expected number of events as given by the model. Using various probability cut-offs, we found that a probability cut-off of 7% (where any probability less than 7% was assigned a value of 0 [non-severe case] and any of 7% and over was assigned a value of 1 [severe case]) correctly classified 26 of the 34 cases (sensitivity 76.4%) and 201 of the 286 non-cases (specificity of 70.3%) and yielded an area under the ROC curve of 0.75 (95% CI: 0.66 to 0.83).

The validation of the model with bootstrapping showed estimates (standard error and bias) with narrow confidence intervals (Table 2). The results of model validation are shown in Table 2.

The centre effect was not significant when included in the logistic regression model, OR 1.8, 95% CI 0.85 to 3.9, comparing social security system to public health, and did not affect the other predictor variables.

## Discussion

We found that four variables, ascites, headache, petechiae, and low platelets on admission, were independently associated with severe dengue. Our logistic regression model could correctly classify 71% of patients when we set the threshold probability for a being a case at 7%, as indicated by the ROC curve. The model was internally validated by using a bootstrap approach.

Ascites has been reported to be present in the very early stages of dengue and has frequently been associated with evolution to more a severe disease stage. This has been previously reported by other investigators, including ascites not only being a predictor for severe dengue but as an early predictor for admission to intensive care [10, 19, 20]. Similarly, a reduction in platelet count to values under 100,000/mm3 has also been reported as an early warning sign for severe dengue [1, 21]. We found that platelets <50,000/mm^3^ were significantly associated with severe dengue in our regression model. Previous reports have described headache as a symptom more likely to be associated with non-severe dengue and occurring with other symptoms like ocular pain [22, 23], consistent with the negative association found in our study.

The presence of petechiae was negatively associated with severe dengue in our model. One previous study found petechiae to be associated with severe dengue, however the comparator was of patients without dengue [8]. Another study found rash to be associated with severe dengue and as well as low platelets (< 30,000/ mm^3^) but did not report petechiae [9]. Although there was a high prevalence of petechiae in patients with severe dengue in a third study, there was no comparison made to non-severe dengue cases [12].

Our model differs from previous ones in that we used very few laboratory indicators. Prior models that have been developed for severe dengue have used different combinations of laboratory variables (e.g.white blood cells count, aspartame aminotransferase). However, higher cut-off points for thrombocytopenia have been included in previous studies as well as petechiae as a positive predictor [4, 11, 12]. Our study sample consisted of patients with dengue fever admitted to a health facility. Our study differs from many others in that it included information available in the first few hours of admission, using data being collected as medical personnel were triaging the patients.

A strength of our study is that the model equation was transformed into a score that could be applied to patients to classify them as severe cases or non-severe cases. Validation of the model, preferably in a different study sample, is an integral part of developing a model. Although lack of external validation is a limitation of our model, our use of bootstrapping sampling led to the development of accurate estimators. We acknowledge the fact that death could not be used as an outcome because of the low death rate and so we selected difference of hematocrits in the first two readings after the admission of patients. We acknowledge that using changes in platelet levels would be an alternative approach [3]. We also acknowledge that our use of a positive IgM represents probable and not confirmed dengue and that hydration and volume expansion could have had an effect on hematocrit.

A limitation of our study is that our model yielded a high level of specificity but relatively low sensitivity using a 50% threshold, in a population where the severe cases represented close to 10% of the sample. We reduced the cut-off to 7% for the probability of being a case in order to increase sensitivity, but this inevitably led to a decrease in specificity. Since clinical practice is centered on the identification of cases with high risk, we reasoned that it was important to increase the ability of the model to rule out severe dengue detect, which means that a reduction of the cutoff point may detect a larger number of true cases (about 80%) but also increasing the false positive rate (i.e. reducing specificity). Another potential limitation is that our study was conducted when other viruses, such as Chikungunya and Zika, were not circulating. There is a possibility that this would have affected the predictive value of our model. Since we conducted an analysis using a database on suspected dengue, we did not have access to the final diagnosis of non-dengue cases. Another limitation of our model is that predictive accuracy may have been overestimated. In order to fully validate the model, an external data set would be required and the predictive accuracy tested.

Ideally, our model would best be used in conjunction with other diagnostic methods that add their own accuracy to the model. The use of the model equation and ROC can provide a better sense of the best cut-point, or verification of the adequate selection of this threshold. The development of a score system can provide a way to determine which patients are at higher risk of severe dengue, contributing to a better patients triage in hospitals and their emergency rooms.

The findings in our study are relevant to regions with reduced access to diagnostic tests such as imaging technology to detect fluid extravasation. From a public health perspective, our model can be a valuable tool to screen populations during epidemic periods and in areas with high transmission can assist in the classification of cases.
